# Prevalence, risk factors, and racial disparities in probable and possible dementia: the national health and aging trends study^☆^

**DOI:** 10.1016/j.tjpad.2026.100676

**Published:** 2026-09-12

**Authors:** Young-Shin Lee, Juan A. Cruz, Michelle Kabakibi, Alison Moore, Jung-Ah Lee, Hee-Jin Jun

**Affiliations:** a School of Nursing, San Diego State University, 5500 Campanile Drive, San Diego, CA, 92182, United States; b School of Public Health, San Diego State University, 5500 Campanile Drive, San Diego, CA, 92182, United States; c School of Medicine, University of California San Diego, 9500 Gilman Drive, MC 0665, La Jolla, CA, 92093-0665, United States; d Sue & Bill Gross School of Nursing, University of California, Irvine, 854 Health Sciences Rd. Irvine, CA, 92697-3959, United States

**Keywords:** Cognitive decline, Probable dementia, Risk factors, Education, Hearing aids, Oral dysfunction, Depression, Anxiety, Racial and ethnic disparities

## Abstract

**Background:**

While global frameworks highlight modifiable risk factors for cognitive decline, evidence on how these risks affect diverse populations remains limited. This study determines the prevalence of probable and possible dementia, identifies primary predictors, and investigates risk variations across racial and ethnic groups in a nationally representative sample.

**Methods:**

Using a sample of 7,574 older adults from the 2023 National Health and Aging Trends Study (NHATS), cognitive status was classified as probable, possible, or no dementia. Survey-weighted multinomial logistic regression models with interaction terms examined conditional effects of age, health conditions, and sensory corrections across demographics.

**Results:**

Advanced age, low education, stroke, falls, and depression/anxiety were associated with higher Odds of possible and probable dementia (Prb-D), while female, vision and hearing aid use lowered Prb-D odds. Hispanic adults aged 85 and older faced a 60.2% predicted probability of Prb-D, compared to 35.1% for Non-Hispanic (NH) White adults. Low education disproportionately increased Prb-D prevalence, especially among Hispanics. Among hearing aid users, NH White and NH Black adults showed lower of Prb-D probability than non-users, whereas Hispanic users showed a higher probability. Chewing or swallowing problems severely escalated Prb-D probability for Hispanic adults (42.2%) compared to NH Whites (19.3%), and depression doubled the overall risk of Prb-D.

**Conclusion:**

The burden of cognitive decline is deeply unequal, proving standardized interventions insufficient for minority older adults. Addressing these disparities necessitates culturally responsive care pathways and the elimination of structural barriers, particularly inadequate insurance coverage for audiology and dental services.

## Highlights

• Hispanic older adults exhibit the highest prevalence of probable dementia.
• Dementia risk factors operate differently across racial and ethnic subgroups.
• Hearing aids paradoxically associate with higher dementia odds in Hispanics.
• Chewing problem and depression are severe dementia risks for minorities.
• Universal dementia prevention guidelines may insufficient for minority older adults.

## 1 Background

As life expectancy in the United States has increased from 68.2 years in 1950 to 79.25 years in 2024, the prevalence of cognitive decline and dementia has risen concurrently [1,2] By 2060, the number of older Americans living with Alzheimer's or a related dementia (ADRD) is anticipated to more than double, growing from an estimated 6.07 million (11.3% of older adults) in 2020 to 13.85 million by 2060 [3]. Currently, dementia is the fifth leading cause of death among individuals over 65, driving a profound loss of functional independence and a reduced quality of life [2,[4], [5], [6]] Furthermore, the global economic burden of dementia care is projected to reach $2.8 trillion by 2030 [2]

Because definitive diagnosis requires neuropathological evidence that is rarely available during life, clinical frameworks classify cognitive decline into distinct stages to guide care [7,8] The primary distinction between the two is functional severity: major neurocognitive disorder interferes with independence in daily activities, whereas mild impairment does not [9,10] This stratification allows clinicians to account for diagnostic uncertainty using medical history, imaging, and lab findings in the absence of absolute neuropathological confirmation [8]

***Racial and Ethnic Disparities in Risk*.** In the U.S., the risk of cognitive decline varies significantly by race and ethnicity, with Non-Hispanic (NH) Black and Hispanic older adults bearing a disproportionate burden. The Chicago Health and Aging Project (CHAP) found dementia prevalence rates of 19% for Black adults and 14% for Hispanic adults, compared to just 10% for White adults aged 65 and older [2] Furthermore, demographic projections indicate that the largest absolute growth in dementia cases over the coming decades will occur among Hispanic and Black populations, alongside women and individuals over 85 years of age [3] Variations in life experiences, socioeconomic factors, and underlying health conditions largely account for these differences [2]

***Modifiable Risk Factors*.** Delaying the onset of ADRD symptoms by just five years could result in a 45% reduction in prevalence by 2050, significantly lowering projected Medicare costs [7] Furthermore, the targeted reduction of key risk factors could lower projected caseloads even further [11] Recent longitudinal data indicate a downward trend in age-adjusted dementia incidence across Western countries, a shift largely attributed to the improved management of these modifiable risk factors [12,13]

The 2024 Lancet Commission highlighted 14 specific risk factors across the life course—including limited education, hearing and vision loss, hypertension, obesity, high cholesterol, diabetes, smoking, alcohol consumption, air pollution, traumatic brain injury, physical inactivity, depression, and social isolation [14] While the Lancet Commission and World Health Organization (WHO) recommendations outline risk factors with substantial supportive evidence, there are still many emerging, potentially modifiable risks for dementia. For example, recent research links cognitive decline to oral health deficits such as chewing or swallowing difficulties,[15,16] poor sleep quality,[17,18] and chronic pain [19,20] However, as highlighted by the 2025 U.S. National Academies’ report, there remains a critical need for rigorous data to establish how these risks operate across specific demographic groups in order to develop targeted, equitable public health strategies [21] Additional research is also needed to disentangle risk factors that are specific to ADRD from those that drive other causes of dementia. Furthermore, despite established global frameworks, a critical gap remains in understanding how these modifiable risk factors differentially impact older adults across diverse racial and ethnic backgrounds.

## 2 Aims

This study **aims** to evaluate the impact of modifiable and non-modifiable risk factors across the spectrum of cognitive decline and to **compare these findings** with internationally recognized risk frameworks.

**Specific aims** are to: 1) Determine the prevalence of Probable, Possible, and No dementia cognitive statuses within a nationally representative sample of older adults; 2) identify the primary predictors of cognitive impairment; and 3) examine conditional interactions to determine how these risk profiles vary across distinct demographic subgroups.

## 3 Methods: measures and analysis

This descriptive study utilized data from 2023 NHATS data, a nationally representative sample of Medicare beneficiaries designed to track late-life disability and cognitive trajectories. This specific 2023 wave was selected because it provides the most current cross-sectional snapshot of cognitive health and includes an increased sample of Hispanic participants (7.8%), enabling a statistically rigorous, representative analysis of these crucial demographic disparities. The sample included 7,574 adults aged 65 and older living independently in the United States.

**Cognitive Classification Methodology.** Because formal clinical diagnosis relies on resource-intensive combinations of medical history, cognitive screenings, imaging, and biomarker testing,[5] ADRD is frequently underdiagnosed [2]

To accurately capture the spectrum of cognitive decline at the population scale, NHATS utilizes a robust, validated algorithm combining prior medical diagnoses, self-reported and proxy-reported functional limitations, and cognitive testing. This methodology allows for a broad, highly accurate classification of "probable dementia" (mirroring the functional impairment of major neurocognitive disorder) and "possible dementia” (mirroring mild neurocognitive disorder/MCI). By utilizing this validated algorithm, NHATS effectively establishes clinical thresholds for cognitive impairment without relying solely on specialized clinical biomarkers [7,22]

**Outcome variable.** The primary outcome variable was cognitive status, categorized into three distinct groups: Probable dementia, Possible dementia, and No dementia, based on the established NHATS classification algorithm. Cognitive impairment was determined using a combination of clinician reports, proxy reports, and self-assessments [23] ***Probable Dementia*** was defined by either (a) a formal clinician diagnosis of any type of dementia; (b) for self-respondents without a formal diagnosis, significant impairment in two or more cognitive domains (memory, orientation, and executive function via the Clock Drawing Test); or (c) for proxy respondents, a score of ≥ 2 on the AD8 (Eight-item Informant Interview to Differentiate Aging and Dementia, score range: 0-8) [24,25] ***Possible Dementia:*** Characterized by impairment in exactly one of the three cognitive domains (for self-respondents) without a formal diagnosis. ***No Dementia:*** Defined as having no formal diagnosis and passing all cognitive screening.

**Modifiable Risk Factors and Covariates.** Independent variables were selected based on risk frameworks from the 2024 Lancet Commission on dementia prevention, the U.S. National Academies of Sciences, the Alzheimer’s Association, and emerging literature [2,[14], [15], [16], [17],[19], [20], [21]] To distinguish established neurological risks from broader markers of physical decline, variables were categorized into specific domains.

The ***primary modifiable risk factors*** included chronic health conditions (hypertension, diabetes, stroke), mental health status (depression/anxiety), and physical and sensory function limitations. These physical and sensory limitations included the use of hearing and/or vision aids, history of falls, sleep difficulties, chronic pain, and chewing or swallowing problems. While not explicitly listed in the Lancet Commission guidelines, limitations such as chewing and swallowing difficulties were analyzed as primary modifiable risk factors-rather than simple covariates-because recent literature identifies them as critical, functional proxies for neuromuscular decline in aging populations [15,16,26]

Baseline demographic variables, including age (categorized as Young-old [65–74], Old [75–84], and Old-old [≥85]), gender, race/ethnicity (NH White, NH Black, and Hispanic), and education (less than high school, high school graduate or GED, some college or associate degree, and bachelor’s degree or higher) were included as covariates in the models to adjust for baseline differences. Except for these categorical demographic variables, all health conditions and physical functional limitations were coded as binary variables (Yes/No).

## 4 Analysis

All statistical analyses were conducted using SAS software and the SURVEYLOGISTIC procedure[27] to account for the complex survey design of the NHATS Round 13 data [28,29] To ensure the results were nationally representative, the models incorporated analytic weights, clusters, and stratification variables provided by NHATS. Taylor series linearization was used as the standard method for variance estimation.

Although the outcome variable (No, Possible, and Probable dementia) follows an ordinal clinical structure, we utilized a survey-weighted multinomial logistic regression model (generalized logit) rather than an ordinal proportional odds model to evaluate the predictors of dementia status [30] This approach was deliberately selected to relax the proportional odds assumption, allowing us to capture the distinct and varying effects of risk factors across different stages of cognitive impairment without constraining the odds ratios to be equal across transition points.

We utilized a three-step modeling process. First, we estimated a main-effects model to assess the independent associations of sociodemographic characteristics and health factors—such as stroke, falls, vision correction, and depression/anxiety—with dementia status. Second, we constructed interaction models to test whether the impact of these factors varied by race and ethnicity. After identifying significant terms in the full model, a final model was fit retaining the statistically significant race/ethnicity interaction with age categories, educational attainment, hearing aid use, and chewing or swallowing problems, and depression/anxiety. Results from the regression models are reported as odds ratios (ORs) with 95% confidence intervals (CIs).

To facilitate the interpretation of these complex interactions, we converted the results into predicted marginal probabilities using the LSMEANS statement with the ILINK and CL options [31] This method allowed us to calculate the model-adjusted predicted probability of each cognitive category for every racial and ethnic group as a percentage, based on the final survey-weighted multinomial logistic regression model. Hereafter, these values are referred to as predicted probabilities. Descriptive percentages were calculated using NHATS analytic weights unless otherwise noted. All statistical tests were two-sided, and significance was evaluated at the alpha = 0.05 level.

## 5 Results

### 5.1 Sample characteristics

Of the 7,574 older adults included in the study, the age distribution was predominantly 65 to 74 years (51.3%), followed by 75 to 84 years (37.3%) and 85 years and older (11.4%). The sample was 44.6% male and 55.4% female. Educational attainment was distributed across bachelor’s degree or higher (34.9%), some college or associate degree (31.4%), high school graduate/GED (22%), and less (11.7%). The racial and ethnic composition was primarily NH White (81.5%), with NH Black and Hispanic older adults comprising 9.3% and 9.2% of the population, respectively (Table 1).

**Table 1:** Demographic and Clinical Risk Factors by Cognitive Status (Survey Weighted Percentages).

*Variable*	Total	No Dementia	Possible Dementia	Probable Dementia	
	*N (%)*	*n (%)*	*n (%)*	*n (%)*	*p-value*
**Total**	7574 (100)	5910 (85.0)	744 (7.2)	920 (7.8)	
**Age**					
65 - <74	2547 (51.3)	2226 (90.5)	189 (5.4)	132 (4.1)	<.001
75 - <85	3350 (37.3)	2682 (84.3)	314 (7.2)	354 (8.5)	
85+	1677 (11.4)	1002 (62.6)	241 (15.3)	434 (22.1)	
**Gender**					
Male	3198 (44.6)	2491 (84.4)	350 (7.8)	357 (7.8)	.251
Female	4376 (55.4)	3419 (85.5)	392 (6.7)	563 (7.8)	
**Race/Ethnicity**					
White	4601 (81.5)	3875 (87.6)	321 (5.9)	405 (6.5)	<.001
Black	1520 (9.3)	1095 (76.5)	183 (11.1)	245 (12.3)	
Hispanic	1453 (9.2)	940 (70.3)	240 (14.5)	273 (15.2)	
**Education**					
BA/BS+	2174 (34.9)	1910 (91.5)	117 (4.1)	147 (4.4)	<.001
Some College	2141 (31.4)	1811 (88.3)	157 (6.2)	173 (5.5)	
HS/GED	1744 (22.0)	1356 (80.8)	165 (8.2)	223 (11.1)	
<HS	1515 (11.7)	833 (64.5)	305 (17.6)	377 (18.0)	
**Hypertension**					
No	2193 (35.7)	1774 (86.5)	192 (6.7)	227 (6.8)	.078
Yes	5381 (64.3)	4136 (84.1)	552 (7.5)	693 (8.4)	
**Diabetes**					
No	5247 (73.6)	4181 (86.3)	477 (6.6)	589 (7.1)	<.001
Yes	2327 (26.4)	1729 (81.4)	267 (8.8)	331 (9.8)	
**Stroke**					
No	7178 (94.4)	5687 (86.0)	689 (7.0)	802 (7.0)	<.001
Yes	396 (5.6)	223 (66.7)	55 (11.5)	118 (21.8)	
**Falls 2+**					
None/once	6328 (84.3)	5078 (87.0)	598 (6.7)	652 (6.3)	<.001
More than once	1246 (15.7)	832 (74.3)	146 (9.6)	268 (16.1)	
**Hearing Aid Use**					
No	6169 (82.6)	4799 (85.2)	598 (6.8)	772 (8.0)	.082
Yes	1405 (17.4)	1111 (84.1)	146 (8.9)	148 (7.0)	
**Vision Correction**					
No	853 (9.7)	528 (73.4)	121 (11.7)	204 (14.8)	<.001
Yes	6721 (90.3)	5382 (86.2)	623 (6.7)	716 (7.1)	
**Chewing/Swallowing Problems**					
No	6725 (90.3)	5367 (86.0)	628 (6.9)	730 (7.1)	<.001
Yes	849 (9.7)	543 (75.9)	116 (9.6)	190 (14.5)	
**Bothered by Pain**					
No	3218 (43.0)	2540 (86.1)	301 (6.8)	377 (7.1)	.168
Yes	4356 (57.0)	3370 (84.1)	443 (7.5)	543 (8.3)	
**Sleep Difficulties**					
Rarely/No	3961 (55.0)	3188 (87.1)	331 (6.2)	442 (6.8)	<.001
Yes	3613 (45.0)	2722 (82.5)	413 (8.4)	478 (9.1)	
**Depression/Anxiety**					
No	5291 (72.2)	4383 (88.5)	455 (6.3)	453 (5.2)	<.001
Yes	2283 (27.8)	1527 (75.8)	289 (9.5)	467 (14.6)	

Note: BA/BS+ = bachelor’s degree or higher; Some college includes associate degree; HS/GED = high school graduate or GED; < HS = less than high school.

Bivariate analyses revealed significant differences in cognitive categories (no dementia, possible dementia, and probable dementia) across most demographic and health variables. Specifically, cognitive status varied significantly by age, education and race/ethnicity, as well as by the presence of diabetes, a history of stroke, a history of multiple falls, lack of vision correction, chewing or swallowing problems, sleep difficulties, and depression/anxiety (all *p-values* < .001). Conversely, no statistically significant differences in cognitive status were observed for gender (*p* = .251), hypertension (*p* = .078), hearing aid use (*p* = .082), or being bothered by pain (*p* = .168) at the bivariate level.

### 5.2 Prevalence of cognitive impairment and risk factors

Table 2 presents the results from the multinomial logistic regression model comparing the odds of possible and probable dementia to the no-dementia reference group. The model revealed significant interactions between race/ethnicity and five primary variables: age (*p* = .013), education (*p* = .014), hearing aid use (*p* = .046), chewing or swallowing problems (*p* = .004), and depression/anxiety (*p* = .045).

**Table 2:** Survey-Weighted Multinomial Logistic Regression Model for Possible and Probable Dementia with Significant Race/Ethnicity Interactions.

Variables		Int. p-value†	Possible Dementia			Probable Dementia		
			OR	95% CI	p-value	OR	95% CI	p-value
Age^1^	65-74		1.00			1.00		
	75-84	.013	1.48	(1.06 - 2.07)	.022	2.37	(1.45 - 3.88)	<.001
	85+		4.85	(3.37 - 6.98)	<.001	8.08	(5.32 - 12.26)	<.001
Gender	Male		1.00			1.00		
	Female		0.72	(0.57 - 0.91)	.005	0.77	(0.62 - 0.95)	.017
Race & Ethnicity^2^	White		1.00			1.00		
	Black		1.42	(0.62 - 3.28)	.411	1.79	(0.88 - 3.65)	.110
	Hispanic		1.64	(0.74 - 3.63)	.224	1.41	(0.71 - 2.80)	.325
Education^1^	BA/BS+	.014	1.00			1.00		
	Some College		1.48	(0.97 - 2.26)	.067	1.12	(0.83 - 1.52)	.461
	HS/GED		2.03	(1.34 - 3.07)	<.001	2.54	(1.79 - 3.61)	<.001
	< HS		3.22	(2.11 - 4.91)	<.001	2.16	(1.33 - 3.51)	.002
Hypertension	No		1.00			1.00		
	Yes		0.84	(0.65 - 1.08)	.177	0.83	(0.65 - 1.05)	.119
Diabetes	No		1.00			1.00		
	Yes		1.10	(0.87 - 1.39)	.417	1.08	(0.85 - 1.37)	.537
Stroke	No		1.00			1.00		
	Yes		1.49	(0.98 - 2.27)	.065	2.50	(1.77 - 3.53)	<.001
Falls 2+	No		1.00			1.00		
	Yes		1.47	(1.10 - 1.97)	.010	2.40	(1.88 - 3.06)	<.001
Hearing Aid Use^1^	No	.046	1.00			1.00		
	Yes		1.25	(0.92 - 1.68)	.150	0.65	(0.45 - 0.95)	.027
Vision Correction	No		1.00			1.00		
	Yes		0.58	(0.42 - 0.81)	.001	0.49	(0.36 - 0.65)	<.001
Chewing/Swallowing^1^	No	.004	1.00			1.00		
	Yes		0.90	(0.58 - 1.40)	.656	1.16	(0.79 - 1.70)	.441
Bothered by Pain	No		1.00			1.00		
	Yes		1.03	(0.81 - 1.32)	.795	0.85	(0.66 - 1.10)	.215
Sleep Difficulties	No		1.00			1.00		
	Yes		1.14	(0.89 - 1.47)	.293	0.94	(0.77 - 1.15)	.576
Depression/ Anxiety^1^	No	.045	1.00			1.00		
	Yes		1.40	(0.99 - 1.98)	.059	2.89	(2.20 - 3.78)	<.001

† Int. (Interaction) p-value represents Type 3 joint test for the interaction between race/ethnicity and the corresponding predictor.

1 Main-effect odds ratios for predictors included in race/ethnicity interaction terms are conditional on NH White adults, the reference race/ethnicity group.

2 Race/ethnicity main effect odds ratios are conditional on reference levels of the interacting predictors: age 65–74 years, BA/BS+ education, no hearing aid use, no chewing/swallowing problems, and no depression/anxiety. Note: BA/BS+ = bachelor’s degree or higher; Some college includes associate degree; HS/GED = high school graduate or GED; < HS = less than high school; Chewing/Swallowing refers to chewing or swallowing problems.

**Fig. 1:**
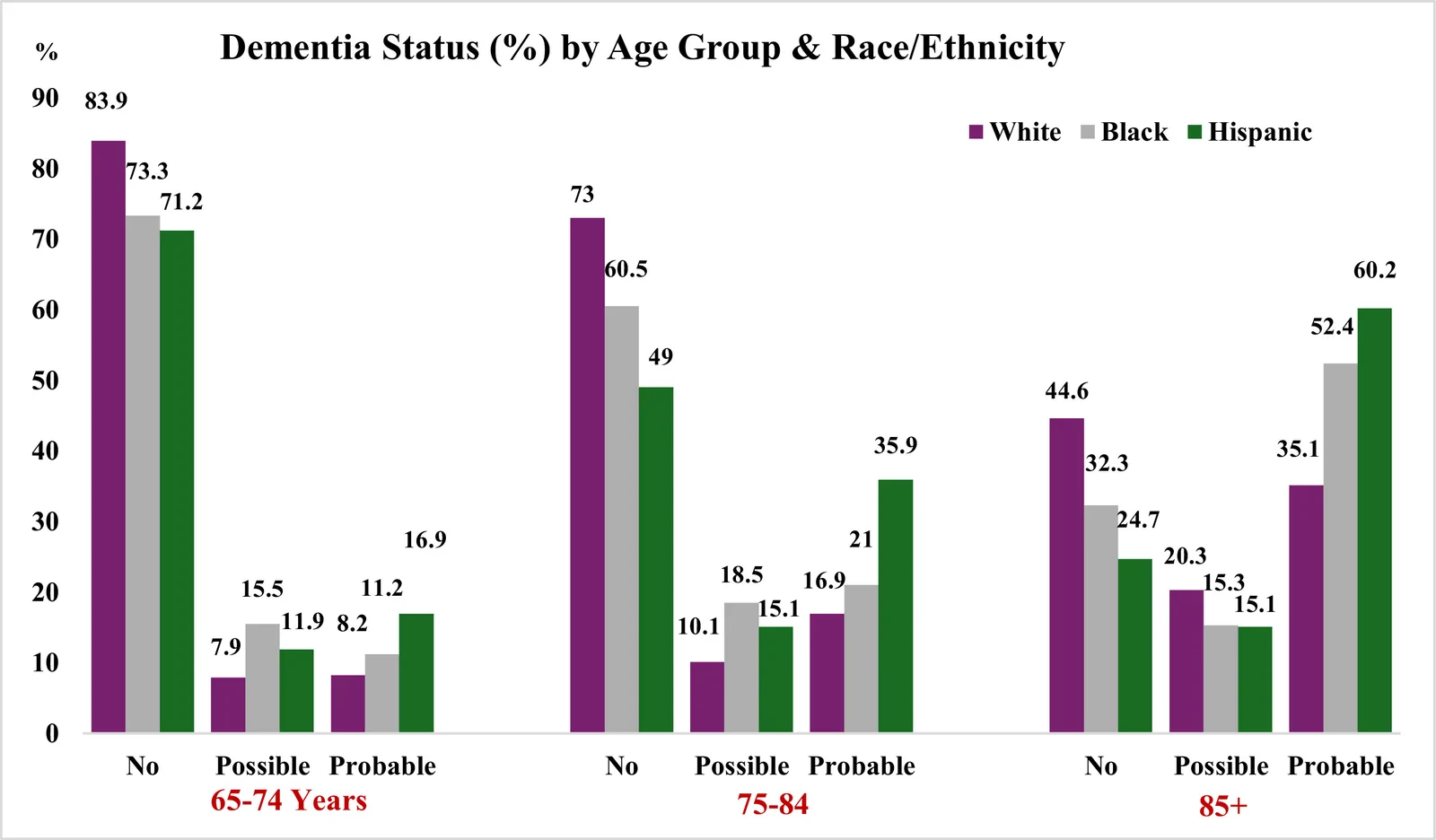
Model-adjusted predicted probabilities of dementia status by race/ethnicity and predictors with significant interaction effects. Dementia Status (%) by Age Group & Race/Ethnicity.

These interactions indicate that the associations between these variables and dementia status differed by race/ethnicity. Therefore, the main-effect ORs for these five variables in Table 2 should be interpreted as estimates for the reference race/ethnicity group, NH White adults. Similarly, the race/ethnicity ORs in Table 2 represent racial/ethnic differences only at the reference levels of the interacting variables: age 65–74 years, BA/BS+ education, no hearing aid use, no chewing or swallowing problems, and no depression/anxiety. To make these interaction findings easier to interpret, we calculated model-adjusted predicted probabilities for each race/ethnicity group and predictor category. These predicted probabilities are presented in Table 3 and Fig. 1.

**Table 3:** Model-adjusted Predicted Marginal Probabilities of Dementia Status by Race/Ethnicity and Predictors with Significant Interaction Effects.

Variable	Category	Race	No Dementia % (95% CI)	Possible Dementia % (95% CI)	Probable Dementia % (95% CI)
Age	65–74	White	83.9 (79.3 – 88.7)	7.9 (4.9 – 10.8)	8.2 (5.2 – 11.2)
		Black	73.3 (64.7 – 81.9)	15.5 (9.1 – 21.8)	11.2 (5.0 – 17.5)
		Hispanic	71.2 (64.8 – 77.5)	11.9 (7.8 – 16.1)	16.9 (11.0 – 22.8)
	75–84	White	73.0 (67.1 – 79.0)	10.1 (7.2 – 13.0)	16.9 (11.9 – 21.8)
		Black	60.5 (47.8 – 73.3)	18.5 (9.6 – 27.3)	21.0 (11.6 – 30.3)
		Hispanic	49.0 (39.2 – 58.7)	15.1 (10.3 – 20.0)	35.9 (25.7 – 46.1)
	85+	White	44.6 (37.3 – 52.0)	20.3 (15.8 – 24.7)	35.1 (29.0 – 41.3)
		Black	32.3 (22.4 – 42.0)	15.3 (7.8 – 22.9)	52.4 (38.3 – 66.6)
		Hispanic	24.7 (17.1 – 32.3)	15.1 (9.1 – 21.0)	60.2 (49.5 – 71.0)
Education	BA/BS+	White	78.8 (74.2 – 83.6)	8.1 (5.7 – 10.4)	13.1 (9.3 – 16.8)
		Black	74.1 (62.7 – 85.5)	9.2 (3.8 – 14.6)	16.7 (5.8 – 27.7)
		Hispanic	66.8 (52.9 – 80.7)	10.4 (5.0 – 15.8)	22.8 (8.7 – 36.9)
	Some College	White	74.7 (68.7 – 80.9)	11.4 (7.0 – 15.7)	13.9 (10.5 – 17.2)
		Black	62.1 (50.8 – 73.5)	16.6 (7.3 – 25.9)	21.3 (10.8 – 31.7)
		Hispanic	51.1 (31.8 – 70.5)	12.9 (6.4 – 19.4)	36.0 (15.6 – 56.3)
	HS/GED	White	61.4 (53.8 – 69.1)	12.8 (8.0 – 17.5)	25.8 (19.2 – 32.4)
		Black	52.9 (42.4 – 63.4)	20.6 (10.9 – 30.3)	26.5 (15.9 – 37.1)
		Hispanic	52.3 (39.7 – 65.0)	13.6 (7.0 – 20.1)	34.1 (18.9 – 49.3)
	<HS	White	59.3 (51.3 – 67.4)	19.5 (13.2 – 25.8)	21.2 (12.8 – 29.6)
		Black	36.7 (23.9 – 49.5)	27.2 (16.3 – 38.1)	36.1 (22.0 – 50.2)
		Hispanic	24.9 (19.1 – 30.6)	24.4 (15.9 – 33.0)	50.7 (39.6 – 61.8)
Hearing Aid Use	No	White	67.4 (61.5 – 73.3)	10.9 (7.8 – 14.0)	21.7 (17.1 – 26.3)
		Black	47.3 (40.7 – 54.1)	18.1 (12.5 – 23.6)	34.6 (27.4 – 41.8)
		Hispanic	52.0 (44.3 – 59.7)	15.6 (11.2 – 20.0)	32.4 (26.1 – 38.6)
	Yes	White	70.8 (64.2 – 77.4)	14.3 (10.4 – 18.1)	14.9 (10.4 – 19.4)
		Black	65.5 (48.8 – 82.4)	16.9 (4.6 – 29.1)	17.6 (4.7 – 30.4)
		Hispanic	44.6 (33.2 – 56.1)	14.9 (7.7 – 22.0)	40.5 (24.9 – 56.1)
Chewing/Swallowing Problems	No	White	69.8 (64.6 – 75.1)	13.3 (9.7 – 16.8)	16.9 (13.4 – 20.3)
		Black	63.9 (56.1 – 71.8)	12.7 (7.5 – 17.8)	23.4 (16.0 – 30.8)
		Hispanic	52.7 (45.2 – 60.2)	16.4 (12.0 – 20.8)	30.9 (23.0 – 38.8)
	Yes	White	68.9 (61.2 – 76.5)	11.8 (7.7 – 16.0)	19.3 (13.5 – 25.2)
		Black	49.4 (35.3 – 63.4)	24.3 (14.1 – 34.6)	26.3 (11.7 – 41.0)
		Hispanic	43.7 (33.4 – 54.2)	14.1 (8.7 – 19.4)	42.2 (30.0 – 54.3)
Depression & Anxiety	No	White	76.6 (71.2 – 81.9)	11.7 (8.0 – 15.3)	11.7 (8.5 – 15.0)
		Black	62.6 (52.5 – 72.7)	18.1 (11.7 – 24.6)	19.3 (10.2 – 28.3)
		Hispanic	52.1 (44.0 – 60.2)	14.8 (10.3 – 19.3)	33.1 (24.3 – 41.9)
	Yes	White	60.4 (53.9 – 66.9)	12.9 (9.4 – 16.4)	26.7 (21.6 – 31.9)
		Black	50.7 (40.1 – 61.4)	17.1 (9.2 – 25.0)	32.2 (20.4 – 43.9)
		Hispanic	44.6 (35.6 – 53.5)	15.7 (10.4 – 21.0)	39.7 (28.6 – 50.8)

**Note**: Values are model-adjusted predicted marginal probabilities and 95% confidence intervals estimated from the final survey-weighted multinomial logistic regression model. Percentages represent the predicted distribution across the three dementia-status categories within each race/ethnicity and predictor category. Note: BA/BS+ = bachelor’s degree or higher; Some college includes associate degree; HS/GED = high school graduate or GED; < HS = less than high school

***Sociodemographic Characteristics:*** Advanced age, lower educational attainment, and race/ethnicity were important predictors of cognitive impairment. Although gender was not a significantly associated with cognitive status at the bivariate level, in the adjusted model, female adults had significantly lower odds of possible dementia (OR = 0.72; 95% CI, 0.57–0.91; *p* = .005) and probable dementia (OR = 0.77; 95% CI, 0.62-0.95, *p* = .017) compared to male adults.

Among NH White older adults, those aged 85 and older had higher odds of both possible dementia (OR = 4.85; 95% CI, 3.37-6.98, *p* < .001) and probable dementia (OR = 8.08; 95% CI, 5.32-12.26, *p* < .001) compared to those aged 65 to 74 years. Among NH White older adults, those with less than high school education had higher odds of possible dementia (OR = 3.22; 95% CI, 2.11–4.91; *p* < .001) and probable dementia (OR = 2.16; 95% CI, 1.33–3.51; *p* = .002) compared to those with BA/BS+ education. However, because age and education significantly interacted with race/ethnicity, predicted probabilities are presented in Table 3 and Fig. 1 to show how these patterns differed across NH White, NH Black, and Hispanic older adults.

***Primary Risk Factors:*** Several health conditions were significantly associated with higher odds of cognitive impairment in the adjusted model. A history of stroke was associated with 2.5 times higher odds of probable dementia compared to those without a stroke history (OR = 2.50; 95% CI, 1.77-3.53, *p* < .001). A history of two or more falls were also associated with higher odds of both possible dementia (OR = 1.47; 95% CI, 1.10–1.97; *p* = .010) and probable dementia (OR = 2.40; 95% CI, 1.88-3.06, *p* < .001). Among NH White adults, depression/anxiety was associated with higher odds of probable dementia (OR = 2.89; 95% CI, 2.20–3.78; *p* < .001). Because depression/anxiety also significantly interacted with race/ethnicity, race/ethnicity-specific predicted probabilities are presented in Table 3 and Fig. 1.

***Factors Associated with Lower Odds of Cognitive Impairment:*** Sensory correction was associated with lower odds of cognitive impairment in the adjusted model. Among NH White adults, hearing aid users had 35% lower odds of probable dementia compared to nonusers (OR = 0.65; 95% CI, 0.45-0.95, *p* = .027). Because hearing aid use significantly interacted with race/ethnicity, the predicted probabilities in Table 3 and Fig. 1 provide a clearer comparison across racial and ethnic groups. Vision correction was independently associated with lower odds of both possible dementia (OR = 0.58; 95% CI, 0.42-0.81, *p* = .001) and probable dementia (OR = 0.49; 95% CI, 0.36-0.65, *p* < .001).

***Non-significant Variables:*** After adjusting for covariates, hypertension, diabetes, being bothered by pain, and sleep difficulties were not statistically associated with possible or probable dementia (*p* > .05). Although chewing or swallowing problems were not significantly associated with dementia odds among NH White adults, the interaction between chewing or swallowing problems and race/ethnicity was statistically significant (*p* = .004), indicating that this association differed across racial and ethnic groups.

## 6 Conditional disparities and interaction effects

Table 3 and Fig. 1 present model-adjusted predicted probabilities to illustrate the significant race/ethnicity interactions.

**Fig. 2:**
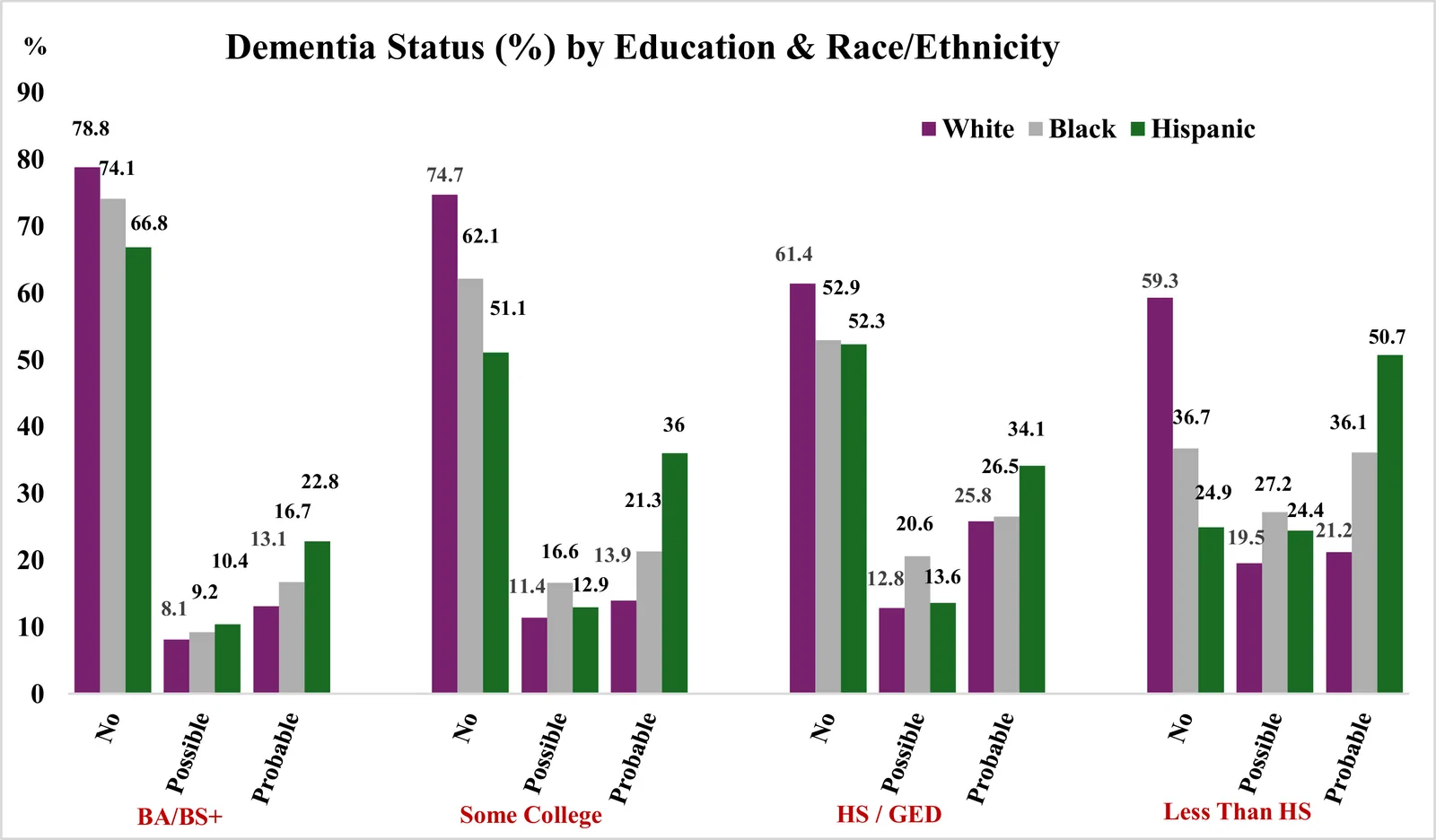
Dementia Status (%) by Education & Race/Ethnicity.

***The Race/Ethnicity-Specific Patterns by Age and Education***: Significant interactions were observed between race/ethnicity and age (*p* = .013) and between race/ethnicity and education (*p* = .014). The predicted probability of probable dementia increased with age across all racial/ethnic groups, but the increase was greater among NH Black and Hispanic older adults (Fig. 1). Among adults aged 85 years and older, the predicted probability of probable dementia was 35.1% for NH White adults, 52.4% for NH Black adults, and 60.2% for Hispanic adults. The predicted probability among Hispanic adults aged 85 years and older was 1.72 times that of NH White adults in the same age group. Lower educational attainment also showed stronger associations with probable dementia among NH Black and Hispanic older adults. Among adults with less than high school education, the predicted probability of probable dementia was 21.2% for NH White adults, 36.1% for NH Black adults, and 50.7% for Hispanic adults (Fig. 2).

**Fig. 3:**
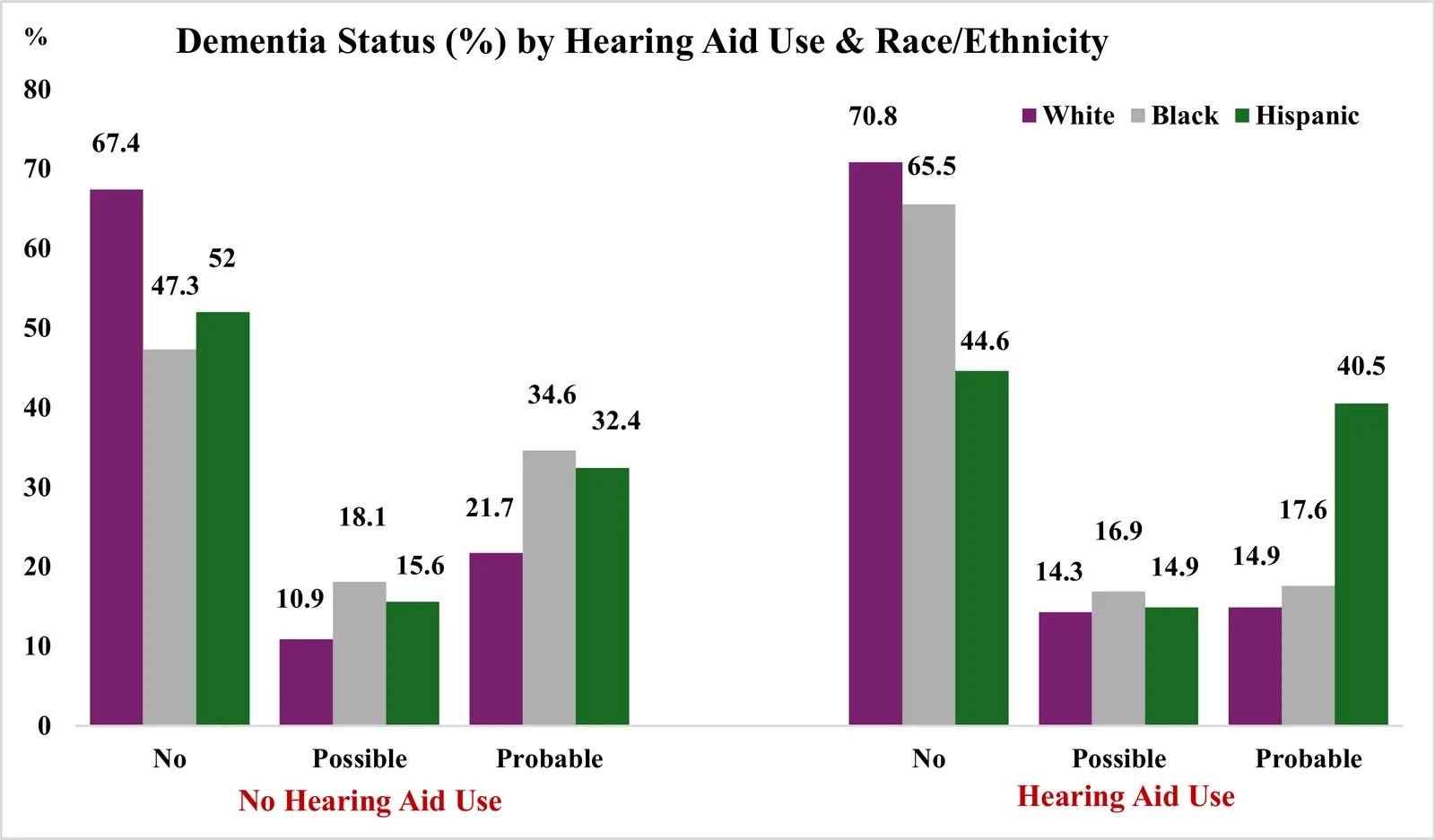
Dementia Status (%) by Hearing Aid Use & Race/Ethnicity.

***Disparities in Hearing Aid Use:*** A significant interaction was identified between hearing aid use and race/ethnicity (*p* < .05) (Fig. 3). Among NH White older adults, hearing aid use was associated with a lower predicted probability of probable dementia (14.9% for users vs. 21.7% for non-users). A similar pattern was observed among NH Black older adults, for whom the predicted probability of probable dementia was 17.6% among hearing aid users and 34.6% among non-users. In contrast, this pattern was not observed among Hispanic older adults. The predicted probability of probable dementia was higher among hearing aid users (40.5%) than among non-users (32.4%). This finding should be interpreted cautiously because hearing aid use does not directly measure the presence, severity, or duration of hearing impairment.

**Fig. 4:**
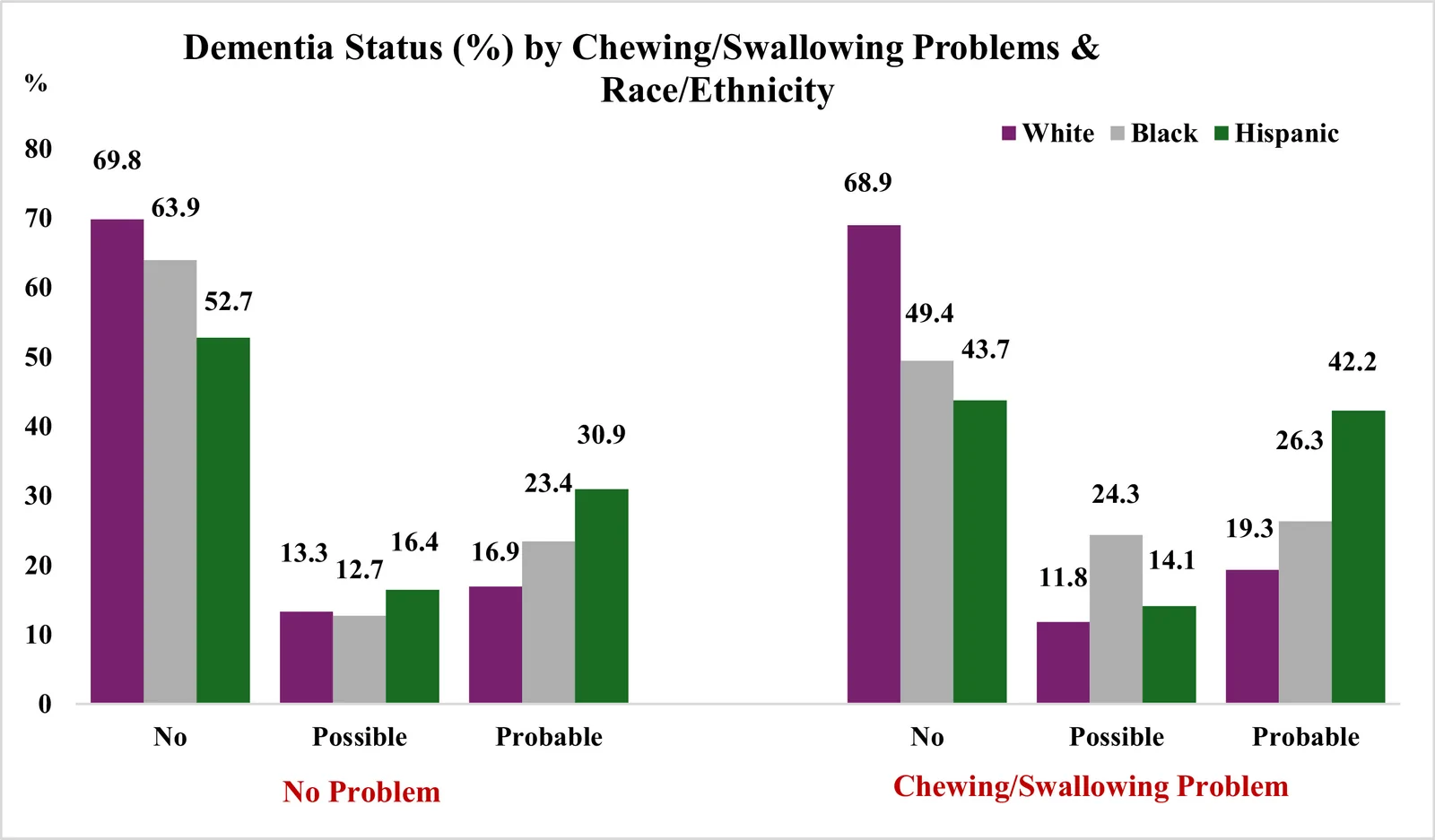
Dementia Status (%) by Chewing/Swallowing Problems & Race/Ethnicity.

***Chewing or Swallowing Problems and Cognitive Disparities:*** Although chewing or swallowing problems were not significantly associated with dementia odds among NH White adults, their interaction with race/ethnicity was statistically significant (*p* = .004, Table 2). For Hispanic older adults, the presence of a chewing or swallowing problem was associated with a higher predicted probability of probable dementia, increasing from 30.9% among those without these problems to 42.2% among those with these problems. For NH Black older adults, chewing or swallowing problems were more strongly associated with possible dementia, with the predicted probability increasing from 12.7% to 24.3%. In contrast, predicted probabilities for NH White adults remained relatively stable by chewing or swallowing status (Fig. 4).

**Fig. 5:**
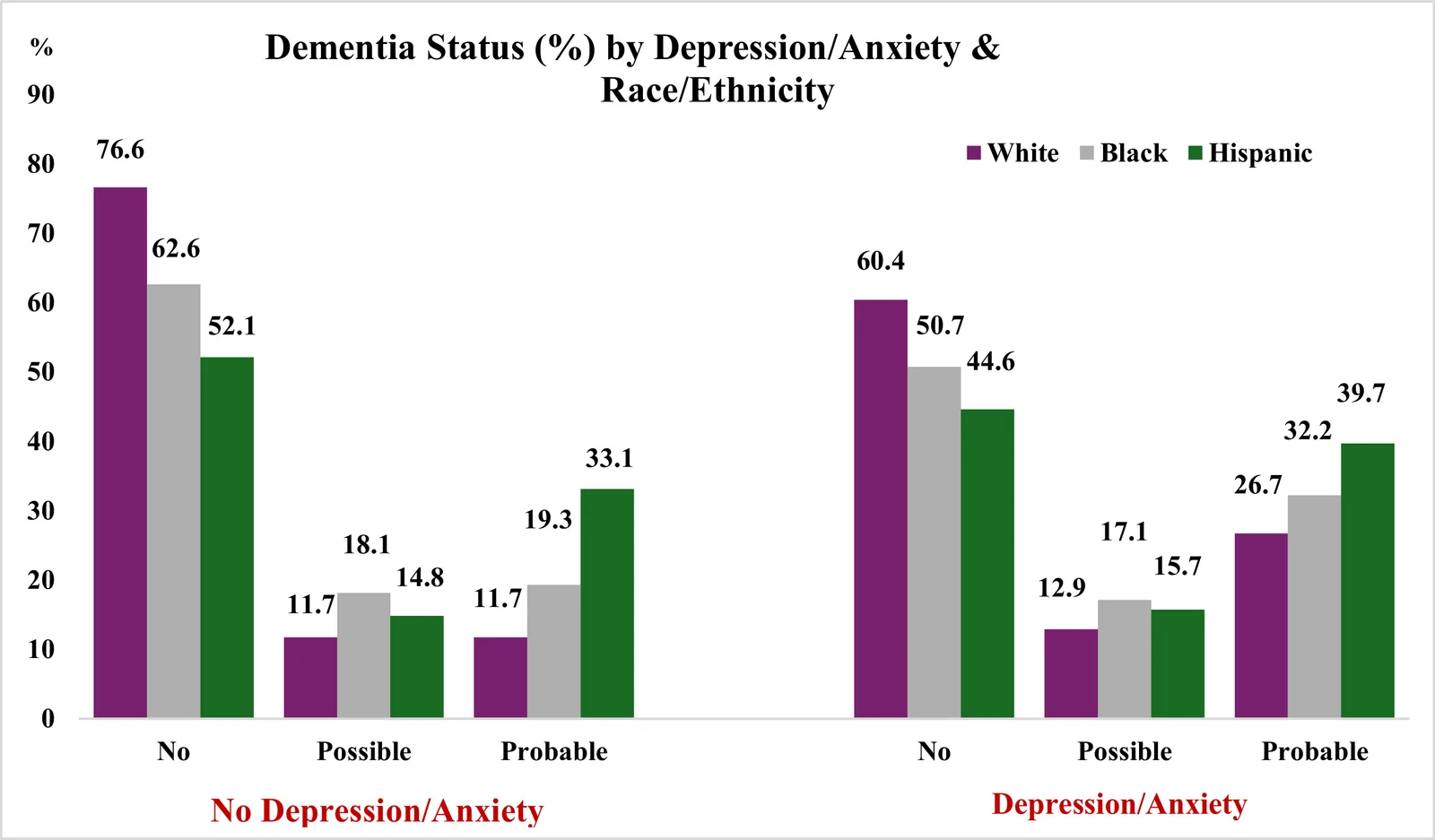
Dementia Status (%) by Depression/Anxiety & Race/Ethnicity. **Note**: Values represent model-adjusted predicted probabilities estimated from the survey-weighted multinomial logistic regression model. Confidence intervals are presented in Table 3.

***Depression/Anxiety:*** Depression/anxiety also significantly interacted with race/ethnicity (*p* = .045). The predicted probability of probable dementia was higher among adults with depression/anxiety than among those without it across all racial and ethnic groups. Among adults with depression/anxiety, the predicted probability of probable dementia was 26.7% for NH White adults, 32.2% for NH Black adults, and 39.7% for Hispanic adults (Fig. 5). In Table 2, the main-effect odds ratio for depression/anxiety reflects the association among NH White adults and shows higher odds of probable dementia (OR = 2.89; 95% CI, 2.20–3.78; *p* < .001), but not possible dementia (OR = 1.40; 95% CI, 0.99–1.98; *p* = .059).

## 7 Discussion

### 7.1 Prevalence and demographic disparities

The current study of U.S. Medicare recipients examined the risk factors for cognitive impairment, building on the 2024 Lancet Commission’s framework of 14 modifiable risk factors across the life course. Using the 2023 NHATS dataset, this analysis provides a contemporary snapshot of cognitive health. In our nationally representative sample, the combined prevalence of possible and probable dementia was 15% (7.2% and 7.8%, respectively). This estimate is slightly higher than the estimates reported in the 2025 Alzheimer’s Association facts and Fig.s [2] Although dementia prevalence did not differ significantly between men and women at the bivariate level, women had lower adjusted odds of possible and probable dementia in the multivariable model, which is consistent with some prior literature [3]

Advanced age and limited education were strongly associated with cognitive impariment, consistent with the Lancet Comission guideline as well as broader literature [[32], [33], [34]] Descriptively, adults aged 85 years and older and those with less than high school education had substantially higher prevalence of possible and probable dementia than their younger or more highly educated counterparts. Prior reports show that dementia risk increases sharply with age, with one study estimating that every 5-year increase in age nearly doubled dementia risk [32] Higher educational attainment has also been consistently associated with lower dementia risk [35]

When stratified by racial and ethnic groups, our study revealed disparities that are more pronounced than those frequently highlighted in prior literature. Hispanic adults aged 65 years and older demonstrated the highest prevalence of possible (14.5%) and probable dementia (15.2%), rates more than double those observed among NH White adults (5.9% and 6.5%, respectively). NH Black adults also had approximately twice the prevalence of possible dementia (11.1%) and probable dementia (12.3%) compared with NH White adults [2]

### 7.2 Clinical risk factors: alignments and contrasts

Regarding health conditions, our bivariate findings align with established literature showing that individuals with diabetes and a history of stroke have a higher prevalence of cognitive impairment [14,36] However, conditions such as hypertension and chronic pain did not show significant differences in dementia prevalence. Furthermore, our findings indicate that individuals experiencing falls, chewing or swallowing problems, and sleep difficulties had significantly higher prevalence rates of possible and probable dementia. Conversely, those utilizing vision correction exhibited a lower prevalence.

In our adjusted multivariable model**,** advanced age, lower educational attainment, stroke, a history of falls, and depression/anxiety were associated with higher odds of probable dementia, although the associations for age, education, and depression/anxiety varied by race/ethnicity. Vision correction was associated with lower odds of cognitive impairment, whereas the association between hearing aid use and probable dementia varied across racial and ethnic groups. These findings partially align with the priorities highlighted by the Lancet Commission and U.S. reports regarding depression/anxiety, hearing-related factors and untreated vision loss [14,37,38] Notably, in contrast to existing literature,[14,36,38,39] our adjusted multivariable model did not show statistically significant associations of hypertension or diabetes with possible or probable dementia, whereas stroke and a history of falls remained strongly associated with probable dementia.

### 7.3 Compounding burden of age, education and race

The most striking findings of this study emerged from the **interaction models**, which demonstrate that the burden of probable dementia differed substantially by racial/ethnicity across age and educational categories. Across the three age groups, Hispanic older adults had 1.7 to 2.1 times the predicted probability of probable dementia compared to NH White adults in the same age group. NH Black adults also had higher predicted probabilities of probable dementia than NH White adults, particularly among those aged 85 years and older. Similar disparities were observed by educational attainment. Among adults with less than high school education, the predicted probability of probable dementia was 50.7% for Hispanic adults and 36.1% for NH Black adults, compared with 21.2% for NH White adults. These findings suggest that advanced age and limited education may be associated with especially high dementia burden among Hispanic and NH Black adults. This pattern may reflect systemic barriers that contribute to delayed or missed clinical dementia diagnosis among Hispanic and NH Black older adults compared with their NH White peers [40]

### 7.4 Untangling the sensory paradox: hearing aids use and race/ethnicity

A central and unexpected finding of this study is the pronounced racial and ethnic variation in the association between hearing aid use and probable dementia. The 2024 Lancet Commission and existing literature identify untreated hearing loss as an important modifiable risk factor for dementia [36,38] In our adjusted model, hearing aid use was associated with 35% lower odds of probable dementia among NH White adults. However, the significant interaction between hearing aid use and race/ethnicity indicated that this association differed across racial and ethnic groups.

For NH White and NH Black older adults, the predicted probabilities followed the expected direction. The predicted probability of probable dementia was lower by 6.8 percentage points for NH White hearing aid users compared with non-users and by 17.0 percentage points for NH Black hearing aid user compared with non-users. However, this pattern was not observed among Hispanic adults. Hispanic hearing aid users had a higher predicted probability of probable dementia than non-users (40.5% vs. 32.4%), and their predicted probability was nearly three times that of NH White hearing aid users (14.9%).

This finding should be interpreted cautiously. Hearing aid use does not directly measure the presence, severity, duration, or adequacy of correction for hearing impairment. One possible explanation is that hearing aids may be adopted later among some Hispanic adults, when hearing loss or cognitive impairment is already more advanced. Consistent with this possibility, Bessen et al. found substantially lower hearing aid use among low-income Hispanic adults (5.3%) than among low-income NH White adults (21.8%) [41] Furthermore, prior longitudinal work by West et al. also suggests that hearing loss, hearing aid use, and cognitive function may be patterned differently across racial and ethnic groups. In that study hearing loss was associated with lower cognitive function across groups, but hearing aid use was not associated with cognitive function among NH Black older adults [42] The same report noted that delays in hearing aid adoption after candidacy averaged nearly 10 years, ranging from 8.6 years among White adults to 15.2 years among non-White adults [42] Thus, in our cross-sectional data, hearing aid use may reflect differences in access, timing of diagnosis, severity of hearing impairment, or adequacy of treatment rather than a uniform association with lower dementia risk.

If hearing aids are adopted only after sensory and cognitive impairments are already more advanced, hearing aid use may function as a marker of delayed care rather than an early sensory correction. Timely identification of hearing loss, appropriate hearing aid fitting, user education, and regular audiologic are therefore critical. Ultimately, these findings suggest that hearing care should not be treated as a uniform intervention across all populations. The presence of a hearing aid alone may not be sufficient if systemic barriers to early screening, optimal fitting, consistent use, and regular follow-up care persist among racial and ethnic minority older adults.

### 7.5 Chewing or swallowing problems

Beyond sensory impairments, our interaction models highlighted that chewing or swallowing problems differentially impact cognitive status across racial and ethnic lines. Although an increased risk of cognitive impairment associated with oral dysfunctions has been reported in the literature [26,43], conditions such as tooth loss, chewing difficulties, dry mouth, and swallowing difficulties were notably absent from the 2024 Lancet Commission’s list of modifiable risk factors.

Our findings suggest that the burden associated with these oral problems is greater among Hispanic and NH Black adults. Among Hispanic adults, the presence of a chewing or swallowing problem was associated with increase in the predicted probability of probable dementia from 30.9% to 42.2%. These predicted probability was more than twice that observed among NH White adults with similar oral dysfunctions (19.3%). Among NH Black adults, chewing or swallowing problems were more strongly associated with possible dementia, with the predicted probability nearly doubling from 12.7% to 24.3%.

While a longitudinal study reported that chewing dysfunction and tooth loss were associated with dementia onset, it did not find a significant association for swallowing problems [26] Evidence suggests that early treatment and prevention of oral problems may be associated with lower dementia incidence [44] However, literature examining the association between oral problems and dementia across specific racial and ethnic groups remains limited. These findings underscore the critical, yet often overlooked intersection of oral health, nutrition, and cognitive function.

Access to dental care may be an important structural contributor to these disparities. Original Medicare generally does not cover routine dental services, leaving many older adults to pay out-of-pocket for dental care. This lack of coverage may disproportionately affect low-income and racial and ethnic minority older adults, allowing minor, treatable oral health problems earlier in life to progress into more severe chewing problems in late life. Integrating geriatric dentistry and nutritional support into standard cognitive care pathways, particularly for underserved populations, may help address this potentially modifiable pathway related to cognitive impairment.

### 7.6 Mental health (depression/anxiety)

In our study, depression/anxiety was associated with higher odds of probable dementia among NH White adults, and the predicted probability of probable dementia was higher among adults with depression/anxiety than among those without depression/anxiety across all racial and ethnic groups. These findings are consistent with the Lancet Commission's frameworks regarding mental health. Prior studies have established that higher depression scores increase the risk of entering a dementia cohort [45] Specifically, depression increases the general risk of dementia (HR= 1.74) and depression occurring at a younger age is associated with an even higher dementia risk (HR=2.07) [46] Furthermore, depressive symptoms have been shown to increase rapidly in the 10 years prior to dementia diagnosis, with depression independently predicting a faster progression to incident dementia [47,48] Notably, Hispanic and Asian older adults with dementia demonstrate a higher hazard of progression to cognitive impairment compared to NH White adults [47] Depression is often considered an early warning sign of developing dementia, rather than a direct cause, though the two factors frequently influence one another [48]

### 7.7 Strengths and limitations

This study possesses several notable strengths. First, by utilizing the 2023 NHATS dataset, the findings provide a highly contemporary, nationally representative snapshot of cognitive health within the U.S. Medicare population. Second, notably, the dataset includes a robust sample size of Hispanic older adults, allowing for meaningful and statistically powered comparisons across racial and ethnic groups. Third, the methodological approach rigorously addresses the limitations of brief cognitive screenings by incorporating both proxy reports (AD8) and performance-based executive function tests (such as the Clock Drawing test), offering a more clinically nuanced classification of "possible" and "probable" dementia. Finally, the application of a survey-weighted multinomial logistic regression model with interaction terms allowed us to identify important racial and ethnic disparities that would have been obscured by a standard main-effects analysis.

Despite these strengths, several limitations must be acknowledged. *Cross-Sectional Design*: The cross-sectional nature of the current analysis precludes the establishment of causality or temporality. For example, we cannot determine whether the observed associations between sensory corrections, such as hearing aid use or vision correction, and dementia status. These associations may reflect potential benefit of sensory corrections, such as hearing aid use or vision correction, reverse causation, or differences in access to care or other unmeasured factors. In addition, hearing aid use and vision correction indicate the use of sensory correction but do not directly measure the presence, severity, duration, or adequacy of correction for hearing or vision impairment. The non-user group may include both individuals who do not need correction and individuals who may need correction but are not using it. *Outcome classification:* Possible and probable dementia were classified using NHATS cognitive measures and proxy reports rather than clinical diagnoses, neuroimaging, or biomarker-based assessments. Therefore, some misclassification of cognitive status is possible. *Self-Report and Proxy Bias:* Although our cognitive outcome measures were robust, several covariate health conditions, such as sleep difficulties, pain, fall history, hearing aid use, vision correction, and chewing or swallowing problems, relied on self-reported or proxy-reported data, which are inherently susceptible to recall bias or reporting differences. *Unmeasured Confounding*: While the models adjusted for a comprehensive set of sociodemographic and clinical factors, unmeasured social determinants of health likely play a substantial role in the observed disparities. Factors such as educational quality, income and wealth, environmental exposures, healthcare access, quality of care, and social support were not fully captured, but may be important for understanding the higher dementia burden observed among Hispanic and NH Black adults.

### 7.8 Conclusion and public health implications

This study found that advanced age, lower educational attainment, history of stroke, history of falls, and depression/anxiety were associated with higher odds of cognitive impairment, while vision correction was associated with lower odds of cognitive impairment. The association between hearing aid use and probable dementia differed by race and ethnicity. Importantly, these findings from nationally representative data showed that the burden of cognitive impairment was not distributed equally. By using interaction models, our findings identified racial and ethnic disparities that may be obscured in standard main-effects models often. The combined burdens of advanced age, limited education, sensory-related factors, and physical frailty was especially pronounced among Hispanic and NH Black older adults.

These findings carry important public health implications. While internationally recognized frameworks like the 2024 Lancet Commission provide vital guidance on modifiable risk factors, our results suggest that treating these risks through a universal, "colorblind" lens is deeply insufficient. For example, the pattern observed among Hispanic adults, in which hearing aid use was associated with higher rather than lower predicted probabilities of probable dementia, highlights that the presence of an intervention alone may not overcome systemic barriers to early, culturally responsive care. Furthermore, the higher predicted probabilities of dementia associated with chewing or swallowing problems among Hispanic and NH Black adults suggest that oral health related functional problems deserve greater attention in dementia risk framework, which is a glaring omission in current global risk frameworks.

Public health strategies must evolve to address the structural and socio-ecological determinants of these disparities. Mitigating preventable pathways to cognitive decline requires integrating multidisciplinary care—such as geriatric dentistry, nutritional support, and bilingual audiology—into standard cognitive care protocols. Most importantly, policy interventions must dismantle structural barriers to access, such as the routine dental and audiological coverage gaps in Original Medicare. Without targeted, culturally tailored screening and continuous care pathways, standardized interventions will continue to widen the gap in cognitive decline among minority older adults, shifting an unsustainable management burden onto individuals, families, and the broader public health infrastructure.

Data is available at https://www.nhats.org/nhats

## CRediT authorship contribution statement

**Young-Shin Lee:** Writing – review & editing, Writing – original draft, Validation, Supervision, Resources, Project administration, Methodology, Funding acquisition, Conceptualization. **Juan A. Cruz:** Visualization, Validation, Software, Methodology, Formal analysis. **Michelle Kabakibi:** Writing – original draft. **Alison Moore:** Writing – review & editing, Validation. **Jung-Ah Lee:** Writing – review & editing, Validation. **Hee-Jin Jun:** Writing – review & editing, Software, Methodology, Formal analysis, Data curation.

## Declaration of competing interest

The authors declare the following financial interests/personal relationships which may be considered as potential competing interests:

Young-Shin Lee reports financial support was provided by The Centers for Medicare & Medicaid Services (CMS). If there are other authors, they declare that they have no known competing financial interests or personal relationships that could have appeared to influence the work reported in this paper.
